# Chitin Biosynthesis Inhibition of *Meloidogyne incognita* by RNAi-Mediated Gene Silencing Increases Resistance to Transgenic Tobacco Plants

**DOI:** 10.3390/ijms21186626

**Published:** 2020-09-10

**Authors:** Vimalraj Mani, Chinreddy Subramanyam Reddy, Seon-Kyeong Lee, Soyoung Park, Hyoung-Rai Ko, Dong-Gwan Kim, Bum-Soo Hahn

**Affiliations:** 1 Department of Agricultural Biotechnology, National Institute of Agricultural Sciences, Rural Development Administration, Jeonju 54874, Korea; vimalraj08@gmail.com (V.M.); suumani@gmail.com (C.S.R.); lsk220@korea.kr (S.-K.L.); psy0203@korea.kr (S.P.); 2 Crop Protection Division, National Institute of Agricultural Sciences, Rural Development Administration, Wanju 55365, Korea; reachsg@korea.kr; 3 Department of Bio-Industry and Bio-Resource Engineering, Sejong University, Seoul 05006, Korea; kimdg@sejong.ac.kr; 4 National Agrobiodiversity Center, National Institute of Agricultural Sciences, Rural Development Administration, Jeonju 54874, Korea

**Keywords:** root knot nematode, *Meloidogyne incognita*, chitin biosynthesis, RNA-interference, transgenic plants

## Abstract

*Meloidogyne incognita* is a devastating plant parasitic nematode that causes root knot disease in a wide range of plants. In the present study, we investigated host-induced RNA interference (RNAi) gene silencing of chitin biosynthesis pathway genes (chitin synthase, glucose-6-phosphate isomerase, and trehalase) in transgenic tobacco plants. To develop an RNAi vector, ubiquitin (UBQ1) promoter was directly cloned, and to generate an RNAi construct, expression of three genes was suppressed using the GATEWAY system. Further, transgenic *Nicotiana benthamiana* lines expressing dsRNA for chitin synthase (CS), glucose-6-phosphate isomerase (GPI), and trehalase 1 (TH1) were generated. Quantitative PCR analysis confirmed endogenous mRNA expression of root knot nematode (RKN) and revealed that all three genes were more highly expressed in the female stage than in eggs and in the parasitic stage. In vivo, transformed roots were challenged with *M*. *incognita*. The number of eggs and root knots were significantly decreased by 60–90% in RNAi transgenic lines. As evident, root galls obtained from transgenic RNAi lines exhibited 0.01- to 0.70-fold downregulation of transcript levels of targeted genes compared with galls isolated from control plants. Furthermore, phenotypic characteristics such as female size and width were also marginally altered, while effect of egg mass per egg number in RNAi transgenic lines was reduced. These results indicate the relevance and significance of targeting chitin biosynthesis genes during the nematode lifespan. Overall, our results suggest that further developments in RNAi efficiency in commercially valued crops can be applied to employ RNAi against other plant parasitic nematodes.

## 1. Introduction

*Meloidogyne incognita* is a notorious obligate endoparasite that causes root knot disease in economically important crops. It severely affects many food and commercial crops and causes a global annual yield loss of 12.3% and 157 billion dollars [1]. The use of methyl bromide-like fumigants is a commonly practiced method of control but because of adverse environmental effects, sustainable new strategies are being developed [2]. The life cycle of *M*. *incognita* starts with hatching eggs in soil and is completed in host roots within 30–45 days. From the soil to plant root, it migrates through specialized structures, such as the stylet, amphids, and esophageal glands, by secreting cellulases through the J2 stage [3]. Furthermore, *M*. *incognita* secretes effector proteins that usurp the root system and divert water and nutrients, resulting in gigantic cells or root knots [4]. Feeding root-knot nematodes (RKNs) become sedentary and further developmental stages (J3, J4, and the female stage), are carried out. Females remain on the root and lay eggs, whereas males will reach out from the root.

Since its discovery in 1994, RNA interference (RNAi) has been an integral part of the unraveling of nematode functional genomics [5]. Disruption or knockdown of mRNA using RNAi affords multiple benefits compared with conventional methods of control [6]. RNAi is sequence-specific, and thus will only target a particular sequence and avoids non-targets. It is also possible to target multiple genes with a single sequence when it is conserved among families [7,8]. Many researchers have employed RNAi in various organisms, including *Caenorhabiditis elegans* (dsRNA) [9], *Drosophila* [10], plants [11], and fungi [12]. In plant parasitic nematodes (PPNs), the first RNAi experiment was carried out using the soaking method. This method has been followed by many researchers [13,14,15,16,17] to induce host-mediated gene silencing [6,18,19,20,21,22,23] to regulate nematode infestations. Although this method is effective, it depends on a number of factors, including dsRNA concentration, length, incubation, and nematode species [24]. To overcome these obstacles, research has led to the in planta RNAi approach, which has successfully disrupted nematode gene expression. Due to the limited availability of candidate resistance genes and the sterility of some crops that impair the progress of conventional breeding, a transgenic approach to nematode resistance appears to be a compelling alternative [25]. In this context, numerous studies have shown that PPNs are susceptible to RNAi and host-induced gene silencing. Several studies achieved successful inhibition of *M*. *incognita* propagation by host-mediated gene silencing in tobacco [20,26,27], *Arabidopsis thaliana* [18,28,29], *Vitis vinifera* [30], and potato [22].

Chitin is abundant in nature and found in many living organisms, including yeast, protozoans, arthropods, and nematodes [31]. The long unbranched polymer chain of chitin is formed by chains of β-1,4-linked residues of *N*-acetyl glucosamine, and chitin plays a crucial structural role in insects, fungus, nematodes, and many other invertebrates. Chitin is mostly located in eggshells, including those of plant and animal parasitic nematodes [32,33,34]. Eggs are surrounded by an eggshell, and the strength of this shell is provided by a chitinous layer [35]. Several enzymes are involved in the chitin biosynthesis pathway: trehalase converts trehalose to glucose, which is ultimately converted into hexokinase. Another crucial enzyme in this pathway is glucose-6-phosphate isomerase (GPI), which converts glucose to fructose-6-phosphate. The last enzyme, chitin synthase (CS), converts UDP *N*-acetyl glucamine to chitin [36].

The aim of this study was to disrupt chitin biosynthesis pathway genes endogenously in the nematode via tobacco-mediated gene silencing. The production of transgenic plants depends on several factors, including culture conditions, cell optimization, and suitable choice of transfer method. The use of a suitable promoter is also important. Although raising transgenic tobacco plants is standard nowadays, it is important to select an appropriate promoter for the gene of interest [37,38,39]. While numerous studies have used the cauliflower mosaic virus CaMV 35S promoter, we employed the *Arabidopsis* ubiquitin promoter [40]. Overall, we disrupted three major *M*. *incognita* chitin biosynthesis enzymes, namely, trehalase, GPI, and CS, using RNAi technology. Transgenic *Nicotiana benthamiana* lines expressing dsRNA were employed to control nematode infection and the effects are described.

## 2. Results

### 2.1. In Silico Mining and Cloning of Chitin Biosynthesis Pathway Genes

The biosynthetic pathway of chitin synthesis is composed of eight enzymes: trehalase, hexokinase, GPI, glutamine-fructose-6-phosphate aminotransferase, glucosamine-6-phosphate *N*-acetyltransferase, phosphoacetylglucosamine mutase, UDP-*N*-acetylglucosamine pyrophosphorylase, and CS. We focused on three major enzymes (trehalase 1, GPI, and CS) in this study. The protein sequences of trehalase 1, GPI, and CS were used for homology analysis. The predicted biosynthesis pathway of chitin in *M*. *incognita* is presented in Supplementary Figure S1 [41]. A list of proteins is presented in Supplementary Tables S1 and S2 with the representative number of gene paralogs in *C*. *elegans*.

**Figure 1:**
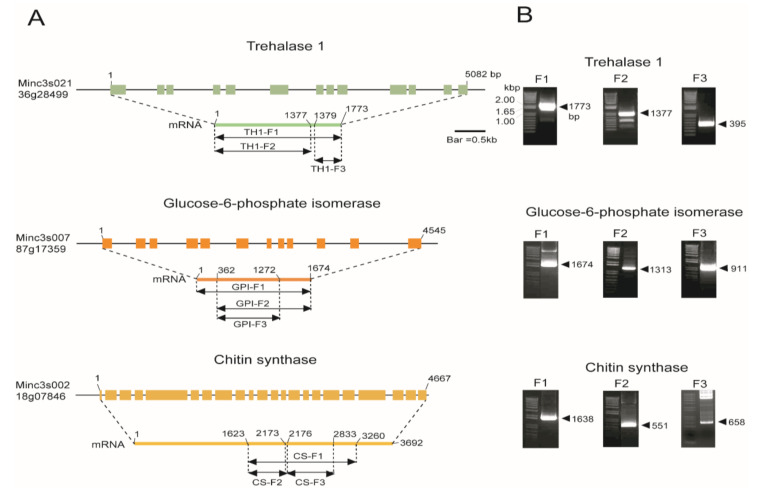
(**A**) Schematic representation of the *Meloidogyne incognita* genome structure of chitin biosynthesis pathway genes. The three major genes of chitin biosynthesis were predicted from the *M*. *incognita* genome as follows: Trehalase 1 (Minc3s02136g28499), glucose-6-phosphate isomerase (GPI) (Minc3s00787g17359), and chitin synthase (CS) (Minc3s00218g07846). (**B**) PCR amplification of different constructs of the three genes (TH1-F1, 1.7 kb; TH1-F2, 1.3 kb; TH1-F3, 0.3 kb; GPI-F1, 1.6 kb; GPI-F2, 1.3 kb; GPI-F3, 0.9 kb; CS-F1, 1.6 kb; CS-F2, 0.5 kb; CS-F3, 0.6 kb) were isolated from *M*. *incognita* and cloned into the PCR^TM^8/GW/Topo^TM^ vector.

Trehalase was found to be present in three copies in the *Meloidogyne incognita* genome, these were named trehalase 1, 2, and 3 (Supplementary Table S1). Trehalase 1 consists of 5082 bp of total gene sequence, 13 exons, and 1773 bp of cDNA. We analyzed the function of trehalase 1 (TH1) fragments in addition to full-length fragments, i.e., TH1-F1, TH1-F2, and TH1-F3. TH1-F1 is a full-length fragment (1773 bp) of trehalase 1, TH1-F2 is an N-terminal fragment (1377 bp), and TH1-F3 is a C-terminal fragment (395 bp). The cloning strategy is depicted in Figure 1 and the primers used are provided in Table 1. Another important gene in this pathway is GPI. The gene structure of GPI was predicted from the *M*. *incognita* genome, and the total GPI sequence contains 4545 bp. It was found to be present in a single copy in the genome and contains 12 exons and 1674 bp. GPI fragments were also cloned: GPI-F1, full-length cDNA (1674 bp), GPI-F2, a C-terminal fragment (1313 bp), and GPI-F3, the middle portion of the cDNA (911 bp). The full-length CS gene is 4667 bp, which includes 3692 bp of cDNA. Three fragments of middle segments of the cDNA were cloned (CS-F1 (1638 bp), CS-F2 (551 bp), and CS-F3 (658 bp)), to analyze the effects of CS (Figure 1). 

Trehalase was found to exist as three different isoforms in the *M*. *incognita* genome and its representative genes were predicted to be type I (Minc3s00333g10409), type II (Minc3s02136g28499), and type III (Minc3s02136g28499). Two hexokinase genes were identified, namely type I (Minc3s00088g04155) and type II (Minc3s00699g16252 and Minc3s01353g23041). Intermediate enzymes (GPI, UDP-*N*-acetyl glucosamine pyrophosphorylase, and phosphoacetyl glucosamine mutase) were present as single proteins in the genome (Minc3s00787g17359, Minc3s05172g37731, Minc3s00584g14658, and Minc3s06387g39757). Glucosamine-6-phosphate *N*-acetyltransferase was found to be present as a single copy (Minc3s02171g28677). Glutamine: Fructose-6-phosphate aminotransferase and CS were found to exist as paralogs in *M*. *incognita* (Minc3s01527g24500, Minc3s03033g32504, Minc3s00218g07846, Minc3s01800g26401, and Minc3s0226g28968). In silico analysis is shown in Supplementary Table S1.

### 2.2. Plant Transformation Vector Construction and Tobacco Transformation

**Figure 2:**
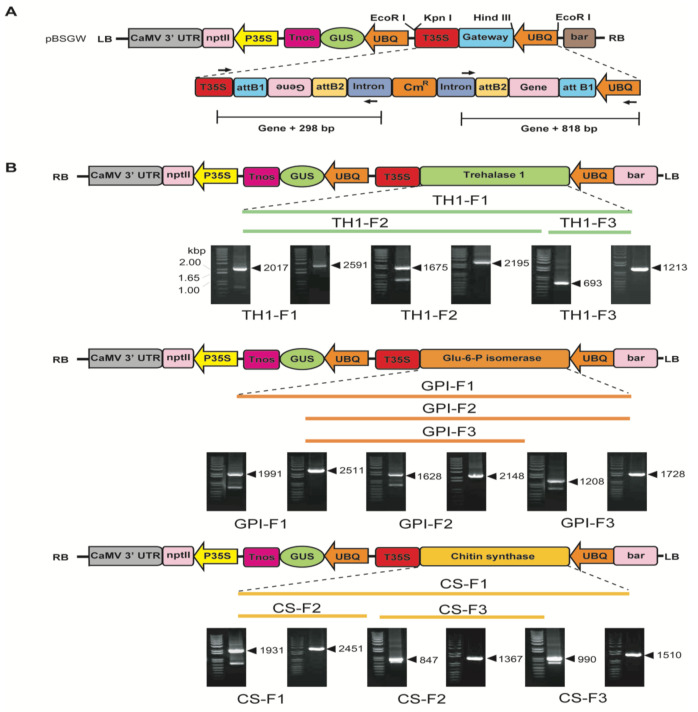
Chitin biosynthesis pathway genes for RNAi vector construction. (**A**) Schematic representation of the vector intended for tobacco transformation. The GATEWAY cassette composed of two recombination sites (attB1 and attB2, two intron sequences, a chloramphenicol-resistance gene (Cm^R^), and the ccdB gene). (**B**) The expression cassette is located between the left and right T-DNA boarders (LB and RB, respectively) and contains two marker genes, a reporter gene along with the desired gene (kanamycin), phosphinothricin-resistance gene (bar), and GUS. Expression of the selection markers and GUS was under the control of the cauliflower mosaic virus 35S promoter and the *Arabidopsis* ubiquitin promoter (UBQ), respectively. Along with selection and reporter genes harboring CaMV 3′ UTR and nopaline synthase terminators, the desired cDNAs trehalase 1, GPI, and CS were under the control of the *Arabidopsis* ubiquitin promoter with the 35S terminator.

To construct the PCR^TM^8/GW/Topo^TM^ vector, partial fragments and full-length cDNAs (trehalase 1, GPI, and CS) were sub-cloned into a gateway plant transformation vector. The primers used for PCR^TM^8/GW/Topo^TM^ and the destination vector cloning are provided in Table 2. An RNAi cassette facilitating chitin biosynthesis genes was constructed using the GATEWAY vector system (Figure 2A). The chitin biosynthesis genes were subcloned into attB1 and attB2 in the GATEWAY vector system and driven by the ubiquitin promoter. Extra sequences in the vector revealed the expected 298 and 818 bp sequences from the intron region and 35S terminator respectively, in the GATEWAY vector (Figure 2A). Figure 2B shows the amplified products along with cDNA sizes and extra sequences in the vector. Briefly, PCR analysis of transgenic plants revealed the expected cDNA product sizes: trehalase 1 (TH1-F1, 2017 and 2591 bp; TH1-F2, 1675 and 2195 bp; TH1-F3, 693 and 1213 bp), GPI (GPI-F1, 1991 and 2511 bp; GPI-F2, 1628 and 2148 bp; GPI-F3, 1208 and 1728 bp), and CS (CS-F1, 1931 and 2451 bp; CS-F2, 847 and 1367 bp; CS-F3, 990 and 1510 bp). The constructed vector was transformed into *Agrobacterium tumefaciens* EHA105 and used for tobacco transformation. GUS and empty vector controls were also transformed into tobacco for further comparison [6].

### 2.3. Chitin Genes Expression Analysis by qRT-PCR

**Figure 3:**
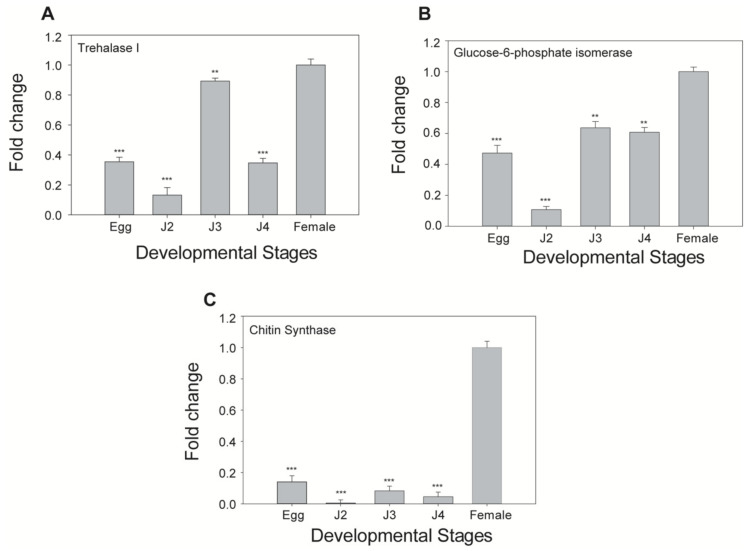
mRNA expression analysis by qRT-PCR at different developmental stages of *M*. *incognita*. Gene expression levels were normalized with β-actin and shown as fold changes in expression compared to the female stage. The results of fold change values were converted using 2^−^^∆∆^^Ct^ values from three genes. (**A**) Trehalase 1 (Minc3s02136g28499), (**B**) GPI (Minc3s00787g17359), and (**C**) CS (Minc3s00218g07846). Each bar represents the mean ± standard deviation (SD) of three independent experiments with three technical replicates. Statistical analyses (*t*-test) were conducted using SigmaPlot 12.5. For *t*-test analysis, female stage values were used to compare to the values of genes in other stages. Asterisk indicates significant differences compared with wild-type (** *p* < 0.01, *** *p* < 0.001).

Quantitative real-time PCR analysis was performed to quantify the endogenous expression of trehalase 1, GPI, and CS in five different developmental stages of *M*. *incognita* (egg, J2, J3, J4, and female stages). The expression levels of trehalase 1, GPI, and CS were relatively higher at the female stage compared with the other four developmental stages of *M*. *incognita*. Therefore, the expression values of these three genes in the female stage were fixed as 1-fold to compare to the values of genes in other stages. Compared with the female stage, trehalase 1, GPI, and CS exhibited 0.14-, 0.47-, and 0.35-fold downregulation in the egg, 0.006-, 0.10-, and 0.13-fold downregulation at J2 stage, 0.083-, 0.63-, and 0.89-fold downregulation at the J3 stage, and 0.04-, 0.60-, and 0.34-fold downregulation at the J4 stage, respectively (Figure 3).

### 2.4. Efficiency of Regeneration and GUS Staining of Transgenic Plants

Using GUS staining, we selected transgenic tobacco plants for further analysis (Supplementary Figure S2A). Putative transgenic lines for all nine constructs were initially screened by PCR with gene-specific primers (Supplementary Figure S2B; Table 1). For each RNAi vector, many transgenic plants were regenerated, and the ratio of GUS-positive stained plants was obtained for each RNAi vector as follows: CS-F1 (32.4%), CS-F2 (43.9%), and CS-F3 (42.9%). The following were obtained with the GPI-silencing RNAi vector: GPI-F1 (32%), GPI-F2 (19.3%), and GPI-F3 (60.5%). In the case of TH1-F1 lines, we obtained only one GUS-positive plant, which did not regenerate further, and this plant was removed from further analysis. The remaining two lines, TH1-F2 (43.6%) and TH1-F3 (62.1%), were included. The overall ratio of regenerated plants and GUS-positive lines is provided in Supplementary Table S3. From each transgenic line, ten independent transgenic plants were used for further propagation; then, the transgenic lines were chosen for nematode suppression analysis.

### 2.5. RKN Inoculation of Transgenic Lines and Suppression Analysis

**Figure 4:**
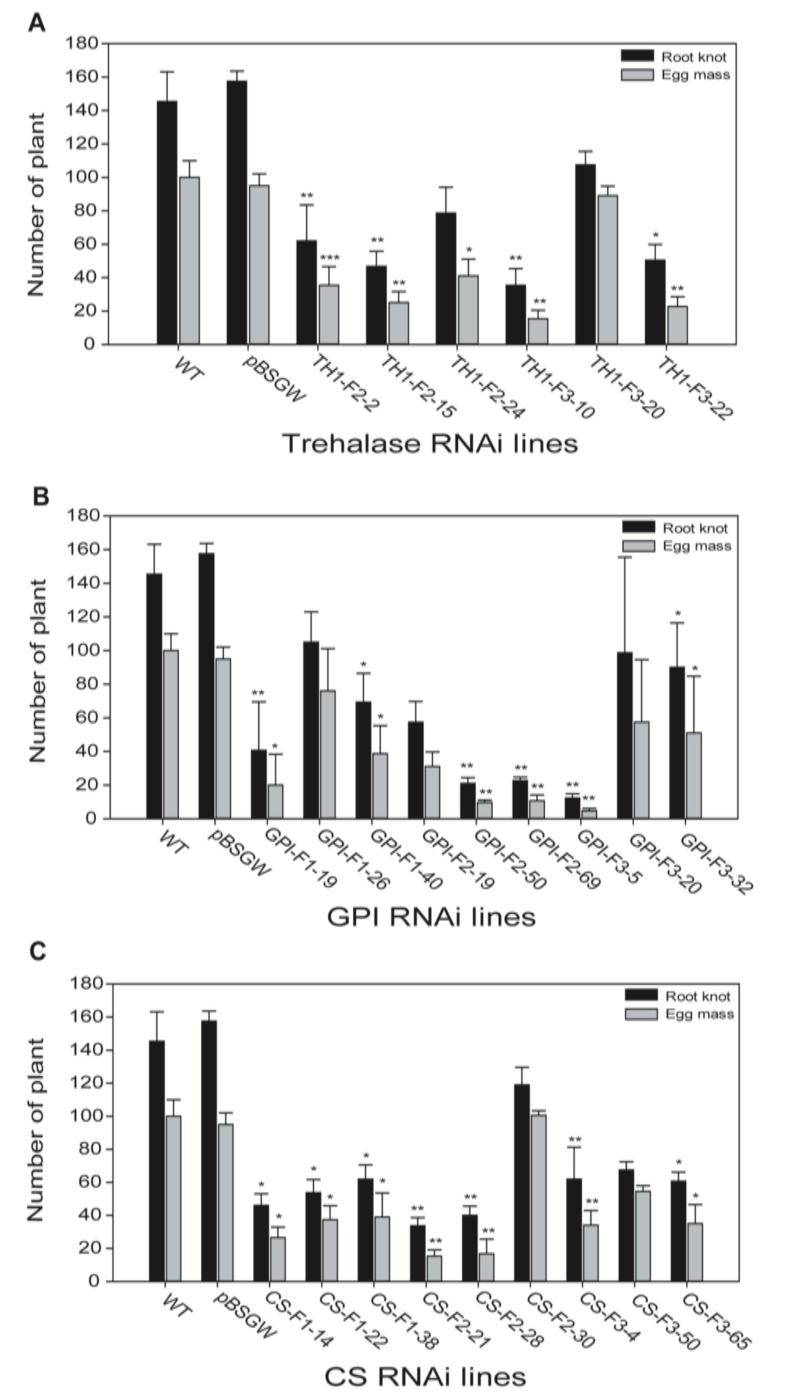
Numbers of root knots and egg masses per RNAi transgenic plant infected with *M*. *incognita*. (**A**) Trehalase 1 RNAi lines, (**B**) GPI RNAi lines, and (**C**) CS RNAi lines. WT, wild type; pBSGW, control vector. Each bar represents the mean ± standard deviation (SD) of three independent experiments with three technical replicates. Statistical analyses (*t*-test) were conducted using SigmaPlot 12.5. For *t*-test analysis, WT data infected by nematode were used to compare with each RNAi line. Asterisk indicates significant differences compared with wild-type, * *p* < 0.05, ** *p* < 0.001, *** *p* < 0.0001.

To elucidate the roles of chitin genes via tobacco-induced gene silencing in *M*. *incognita*, we observed egg mass and root knot numbers in T3 transgenic lines. Root knot numbers ranging from 60 to 100 and egg masses from 20 to 80 were observed in trehalase 1 transgenic lines (TH1-F2–2, TH1-F2–15, TH1-F2–24, TH1-F3–10, TH1-F3–20, and TH1-F3–22) (Figure 4A), while root knot number ranges from 10 to 110 and egg mass numbers from 5 to 80 were observed in GPI transgenic lines (GPI-F1–9, GPI-F1–26, GPI-F1–40, GPI-F2–19, GPI-F2–50, GPI-F2–69, GPI-F3–5, GPI-F3–20, and GPI-F3–32) (Figure 4B). Both trehalase 1 and GPI transgenic lines exhibited less than half the number of egg masses and root knots compared to wild-type and empty vector lines. In the case of chitin transgenic lines, egg mass and root knot numbers were significantly reduced. The root knot numbers of CS-F1–14, CS-F1–22, CS-F1–38, CS-F2–21, CS-F2–28, CS-F2–30, CS-F3–4, CS-F3–50, and CS-F3–65 ranged from 40 to 120, with egg masses of 10–100 per transgenic plant (Figure 4C). However, a significant reduction in the numbers of root knots and egg masses following disruption of chitin synthesis pathway genes was observed: 80% and 75% reductions were observed in TH1-F2–15 and TH1-F3–10 (Figure 4A). The transgenic lines GPI-F2–50, GPI-F2–69, and GPI-F3–5 exhibited 80–90% reduction in egg mass and root knot numbers (Figure 4B). CS transgenic lines (CS-F2–21 and CS-F2–28) showed more than 60% reduction in root knots and egg mass (Figure 4C).

### 2.6. Downregulation of Chitin Biosynthetic Genes of the Female Nematode in RNAi Transgenic Roots

**Figure 5:**
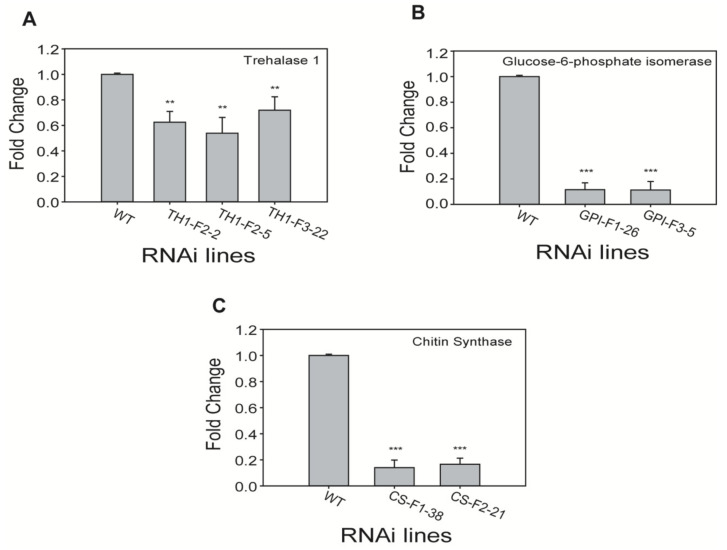
Transcript levels of (**A**) Trehalase 1 (TH1), (**B**) Glucose-6-phosphate isomerase (GPI), and (**C**) Chitin synthase (CS) in root galls of different RNAi transgenic lines infected with *M. incognita*. The difference in gene expression was presented as a fold change relative to the expression of the housekeeping gene (β-actin). Results are presented as mean ± standard deviation (SD) of three independent experiments with three technical replicates. Statistical analyses (*t*-test) were conducted using SigmaPlot 12.5. For *t*-test analysis, the WT plants’ value was used to compare with each RNAi line. Asterisk indicates significant differences compared with wild-type (WT) (** *p* < 0.001, *** *p* < 0.001).

To examine the abundance of target gene transcripts, total RNA extracted from root knots of transgenic plants infected by nematodes was subjected to qPCR. Expression of chitin biosynthetic genes after hairpin-structured RNA ingestion by RKNs was strongly affected. We chose transgenic lines based on the least phenotypical reduction in egg mass and root knot number for qPCR analysis. Expression of trehalase 1, GPI, and CS was strong in infected WT plants, suppressed in infected transgenic plants, and not expressed in uninfected WT plants. While, we examined the gene (Glutathione reductase, Accession number: XM_019390117.1) expressing *Nicotiana benthamiana* plants in uninfected WT plants to check for expression without changes. These results indicated that the three chitin biosynthetic genes in females were actively silenced in their respective RNAi transgenic lines. To determine the relative expression levels of these genes in different plants, their expression in WT plants infected by nematodes were designated as 1-fold. Trehalase 1 expression was downregulated 0.6-, 0.5-, and 0.7-fold in TH1-F2–2, TH1-F2–5, and TH1-F3–22, respectively. Notably, expression levels of GPI and chitin synthase were significantly reduced in their respective transgenic lines: GPI expression was downregulated 0.11-fold in the lines GPI-F1–26 and GPI-F3–5, while CS showed expression levels of 0.14- and 0.16-fold in the lines CS-F1–38 and CS-F2–21, respectively (Figure 5).

### 2.7. Effect of RNAi-Mediated Gene Silencing on Egg Number and Female Morphology in Transgenic Lines

**Figure 6:**
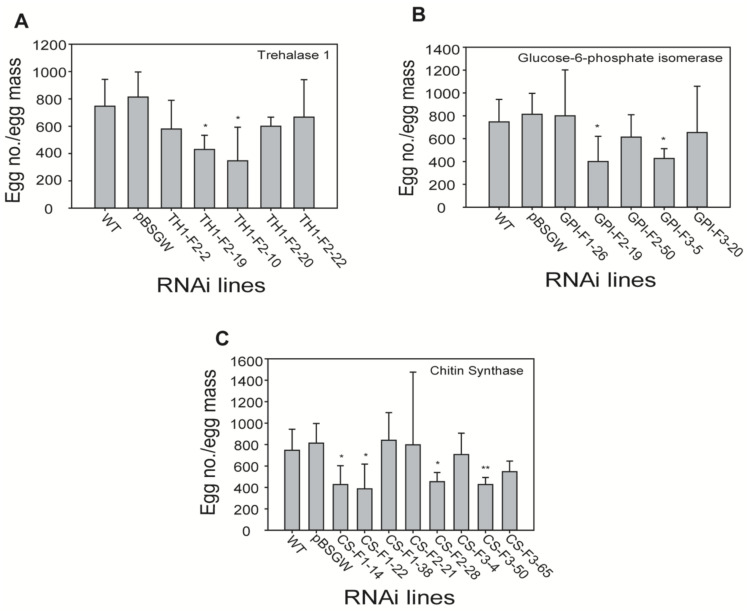
Effect of egg number per egg mass of *Meloidogyne incognita* feeding on transgenic *Nicotiana benthamiana* plants. (**A**) Trehalase 1 RNAi lines, (**B**) GPI RNAi lines, (**C**) CS RNAi lines. Each bar represents mean ± SD of three independent experiments with three technical replicates. Statistical analyses (*t*-test) were conducted using SigmaPlot 12.5. For *t*-test analysis, WT data infected by nematode were used to compare with each RNAi line. Asterisk indicates significant differences compared with wild-type (* *p* < 0.05 and ** *p* < 0.001).

To evaluate the influence of egg number reduction and female morphology in RNAi-mediated gene-silenced transgenic lines, egg number per egg mass of RKN was counted in each RNAi transgenic and control line (wild-type and pBSGW). Egg numbers were statistically reduced in TH1-F2–19, TH1-F3–10, GPI-F2–19, GPI-F3–5, CS-F1–14, CS-F1–22, CS-F2–28, and CS-F3–50 lines (Figure 6). We observed female morphology in the roots of RNAi transgenic and control lines. Interestingly, no anomalies were observed in female morphology in terms of length and width. Female width and length in the control line were 813 and 494 µm, respectively. Thus, female morphology of the transgenic RNAi lines TH1-F2–5, TH1-F3–10, GPI-F1–26, GPI-F2–19, GPI-F3–5, CS-F1–14, CS-F2–28, and CS-F3–50 was similar to the female morphology of the control line. However, significant differences in width were noted in the lines TH1-F2–5, GPI-F1–26, and CS-F1–14. Only one transgenic line (GPI-F2–19) exhibited a decrease in length. Overall, transgenic lines and controls exhibited similar female morphology (Supplementary Figure S3).

## 3. Discussion

The *M*. *incognita* genome [42] has provided an inevitable resource for a better understanding of nematode parasitism factors. RNAi strategy has been exploited well in the elucidation of novel gene functions in a variety of organisms, including the model nematode *C*. *elegans* [9,43]. In this study, the significance of chitin biosynthesis during the life cycle of *M*. *incognita* was investigated by downregulating the three key genes of chitin biosynthesis in transgenic tobacco plants via the RNAi silencing strategy. 

Chitin is a major structural component that supports muscular attachment, and prevents and protects against physical, chemical, and disease damage. The mature eggs were arranged in single female reproductive stage in nematodes and transferred into spermatothera, where fertilization occurs. Eggs in the nematode are therefore usually at different stages of development. The biosynthetic pathway of chitin begins with the conversion of glucose to UDP-GluNAc and culminates in the conversion of UDP-GlcNAc to chitin in insects. Trehalase (an anomer) starts the chitin biosynthesis pathway by hydrolyzing trehalose into glucose. Two kinds of trehalases are usually present in organisms: soluble trehalase located within the cell [44] and membrane-bound extracellular trehalase [36]. The number of paralogs varies in different organisms with different numbers of isoforms, i.e., *C*. *elegans* and *Trichuris murus* have five trehalases, while *B*. *malayi*, *C*. *brenenri*, *C*. *ramanei*, and *S*. *ratti* have 11, 9, 6, and 4 isoforms of paralogous genes, respectively. *Acyrthosiphon pisum* has the highest number of genes (13 isoforms) in insects [45]. Two kinds of trehalases were also identified in the present study (Supplementary Table S1). GPI was found to act as a single copy gene in the *M*. *incognita* genome along with *B*. *malayi* and *C*. *elegans*. Other worms contain more numbers, such as *C*. *brenneri* and *T*. *murus*, which contain four and two isoforms, respectively (https//www.wormbase.org). Chitin synthase was found as two isoforms in *M*. *incognita*, like *C*. *elegans*. Intriguingly, aphid *Acyrthosiphon pisum* contain only a single chitin synthase gene (CHS1) [46]. The CS gene from *M. incognita* seems like a partial one because that contained only nine trans-membrane helices instead of 15 [47,48].

An earlier study examined the effects of RNAi of six genes (*drh-3*, *tsn-1*, *rrf-1*, *xrn-2*, *mut-2*, and *ald-1*) associated with RNAi pathways [49]. In this study, chitin biosynthesis pathway-crucial genes, Trehalase 1, GPI, and Chitin synthase, were used for analysis, hitherto its protein in-silico analysis revealed closely related organisms, like other nematodes. Trehalase 1 (Minc5044) showed high homology to the parasitic nematode *Anisakis simplex* (NCBI Accession No.: AHM26075.1), and free-living nematodes *C. elegans* (NP491890.2) and *Pristinonchus pacificus* (PDM77984). Trehalase 2 (Minc5451) exhibited higher sequence conservation with *Aphelenchoides besseyi* (AKH40415.1), *Brugia malayi* (XP_001900224.1), and *Loa Loa* (XP_003145821.1). GPI sequence showed higher homology to insects, i.e., *Euphydryas viviparis* (ADA56786.1), *Euroglyphus manynei* (OTF8369.1), and *Spodoptera litura* (XP_002818754.1). CS showed proximity to other nematodes, i.e., *Brugia malayi* (AAG49219.1), *Dirofilaria immitis* (AAL92023.1), and *Strongyloides ratti* (XP-24504714). The list of homologs in different organisms is listed in the Supplementary Table S1.

The endogenous expression of three proteases was varied according to the different developmental stages in *M. incognita* [20]. Similarly, serine proteases expression was found to be higher in pre-parasitic juveniles compared with other stages [50]. Fragoso et al. [51] reported that these serine proteases accumulated more abundantly in matured females containing eggs. The present study showed that the expression of trehalase 1, GPI, and CS was maximum in the female stage and minimum in the J2 stage (Figure 3). We assume that the distinct expression of those three genes in different developmental stages of *M*. *incognita* may correspond to the levels of chitin. For example, female sac contains numerous eggs where chitin is abundantly present, whereas J2 stage may contain relatively less chitin.

Many recent studies have applied the RNAi approach to disrupt gene expression in different organisms, including insects [47], fungi [52], and nematodes [53], by host-mediated delivery. In biosafety aspects, RNAi technology for PPN management requires risk assessment and needs proper experimental designs. In addition, avoiding the off-targets effect is an important consideration for all RNAi experiments. Earlier studies reported that off-target silencing of the gene may lead to harmful and adverse effects on plant phenotype [26]. However, we have previously showed that the use of RNAi gene silencing causes no phenotypic effect between the WT and RNAi plants. Similar results were obtained in this study also. In planta delivery, Yadav et al. [20] reported that fragment of RNAi *N. benthamiana* roots successfully silenced two *M. incognita* genes (encoding a splicing factor and an integrase), and results showed significant reduction in gall formation compared to control plants. Many studies have shown that plant-mediated RNAi insertion significantly reduces *M*. *incognita* eggs, egg mass, root knot numbers, and PPN propagation [18,27,28,29,30]. In agreement with this, in this study, the RNAi lines of trehalase 1, GPI, and CS showed 80% and 75%, 80% and 90%, and 55% and 60% reduction in root knot and egg mass numbers, respectively. Silencing of *Mi-cpl-1* and ribosomal proteins caused abnormal phenotypes in female size and fertility [54,55]. In this study, silencing chitin biosynthesis genes marginally altered the female size and width and egg counts in each RNAi line.

Earlier studies reported that the levels of gene expression by nematodes feeding on transgenic roots is a direct molecular evidence of host-derived RNAi-mediated downregulation of target nematode genes by qPCR analysis [6,26,49]. In our qPCR results, it also showed strong expression in the WT-infected plants and slight expression in the RNAi transgenic lines (Figure 5). While not all transformed plant roots actually resulted from a single transformation event, the RNAi effects likely occurred because the extent of RNAi after each transformation depends on position effects. Unsurprisingly, we noticed a different level expression pattern between full length and partial construction of RNAi lines. This indicates that silencing of *M*. *incognita* chitin genes had a major detrimental impact on development and prevented root damage by preventing the formation of gall.

Our results demonstrate that suppression of chitin biosynthesis pathway genes in *M*. *incognita* successfully controlled its propagation by reducing egg mass and egg number but not morphology, possibly because chitin is abundant in eggshells of PPNs. Thus, egg mass and egg number were significantly reduced. Thus, our results show that *N*. *benthamiana* plants can become more resistant to RKNs by silencing genes in the chitin biosynthesis pathway. Overall, our results suggest that this method will be useful in developing RKN resistance in economically important crop plants.

## 4. Materials and Methods

### 4.1. Isolation and Cloning of Chitin Synthesis Genes

Sequences of chitin-related genes (trehalase 1, GPI, and chitin synthase) were derived from analysis of a full-length cDNA library of *M*. *incognita* as reported by Kang et al. [56]. To clone cDNA of each gene, we designed gene-specific primers and isolated the genes using mRNA of the nematode. Trehalase 1 full length (TH1-F1), trehalase 1 *N*-terminal (TH1-F2), and trehalase 1 *C*-terminal (TH1-F3) were amplified. Three GPI cDNA fragments, GPI full length (GPI-F1), GPI *C*-terminal (GPI-F2), and GPI middle part (GPI-F3), and three chitin cDNA fragments, chitin middle part (CS-F1, CS-F2, and CS-F3), were isolated (Figure 2). PCR was performed using three different sets of primers (CS-F1-F and CS-F1-R, CS-F2-F and CS-F2-R, and CS-F3-F and CS-F3-R; GPI-F1-F and GPI-F1-R, GPI-F2-F and GPI-F2-R, and GPI-F3-F and GPI-F3-R; TH1-F1-F and TH1-F1-R, TH1-F2-F and TH1-F2-R, TH1-F3-F and TH1-F3-R) to obtain the CS, GPI, and trehalase 1 cDNA for insertion using the following conditions: 95 °C for 5 min, 94 °C for 30 s, 60 °C for 30 s or 3 min, and 72 °C for 2 min for 40 cycles, and finally 72 °C for 7 min (Table 1). PCR products were cloned into the PCR^TM^8/GW vector using a PCR^TM^8/GW/Topo^TM^ cloning kit (Invitrogen, Calsbad, CA, USA), and fragments of pCR8/GW were cloned into pBSGW using the Gateway LR recombinase (Invitrogen, Calsbad, CA, USA) reaction according to the manufacturer’s instructions.

### 4.2. RNAi Vector Construction

To develop an RNAi vector (pBSGW) with the β-glucuronidase (GUS) gene, p221-UBQ-GUS [6] was digested with *Kpn* I/*Hind* III and treated with T7 DNA polymerase prior to blunt-end ligation. Then, a GUS fragment was amplified by PCR using the flanking primers phosphorylated-gusf1 (Phos-gusf1) and phosphorylated-gusr1 (Phos-gusr1) and ligated into blunt-ended p221-UBQ-GUS. The UBQ1 promoter harboring the GATEWAY cassette was amplified by PCR using three flanking primer sets (RNAi UBQ1 F9 and RNAi UBQ1 R12, RNAi UBQ1 F9 and RNAi UBQ1 R13, and RNAi UBQ1 F9 and RNAi UBQ1 R14). The resulting amplified PCR product contained three additional restriction enzyme sites (*EcoR* I–*Hind* III–*Kpn* I–*EcoR* I) at the end of the UBQ1 promoter and was termed p221-UBQ1. To generate an RNAi construct suppressing expression of chitin biosynthesis genes, the GATEWAY cassette was cloned from pK7GWIWG2 (II) (Invitrogen, Calsbad, CA, USA). pBluescript SK (+) was digested with *Sac* I and treated with T7 DNA polymerase prior to blunt-end ligation. An oligonucleotide containing polycloning sites (MCSF8 and MCSR8) was inserted to add a *Hind* III site, and the resulting vector was termed pBluescript SK-2. The DNA region from attB1 near the CaMV 35S promoter to T35S was prepared by digestion of pK7GWIWG2 (II). First, pK7GWIWG2 (II) was digested with *Apa* I/*Spe* I to obtain the region containing T35S, attR1, ccdB, and attR2, and inserted into pBluescript SK+. The resulting vector was termed pBluescript SK-1. In addition, a *Spe* I digest of pK7GWIWG2 (II) yielded a purified DNA fragment from intron to attR1, which was cloned into pBluescript SK-1. The resulting vector was termed pBluescript SK-2. The RNAi construct was prepared from pBluescript SK-4 following digestion with *Kpn* I/*Hind* III and insertion of the RNAi cassette into p221-UBQ1. The final RNAi vector was termed pBSGW. Successful transfer of the three subcloned cDNA fragments related to chitin biosynthesis into the destination vector was confirmed by DNA sequencing and PCR (Table 3).

### 4.3. Stage-Wise Nematode Sample Preparation

The roots of 14-day-old to 2-month-old *M*. *incognita*-infected tomato plants (*Solanum lycopersicum* var. Rutgers) were harvested from a greenhouse maintained at 25 °C with a 16/8 h day/night period. Stages were identified visually, and samples were collected according to our previously described protocol [57]. Briefly, eggs from the infected roots were washed and treated with 10% NaClO for 5 min, excess water was passed through a 25 µm mesh to collect the eggs, and the eggs were purified using a 35% sucrose gradient centrifugation at room temperature. J2 samples were collected by hatching eggs at 25 °C for 5 days in autoclaved distilled water and samples were finally collected using 5–7 layered KIMTECH Science Wipers on a Petri dish. To collect J3, J4, and female stages, infected roots were washed, chopped, and treated with 7.7% cellulose and 15.4% pectinase followed by washing and filtering through a 75 µm filter. The samples stored on the filter were rinsed in water and nematodes were handpicked using a pipette under a stereomicroscope.

### 4.4. RNA Isolation from Nematode

Total RNA was isolated from all the stages of nematodes as previously described [58]. Frozen nematode samples were ground into a powder and immediately transferred to a 2 mL microcentrifuge tube. A total of 800 μL extraction buffer (200 mM Tris-HCl, 400 mM LiCl, 25 mM EDTA, and 1% SDS, pH 9.0) and 600 μL acidic phenol (pH 4.5) was added and mixed well by placing the tube upright for 2 min. The solution was kept on ice for 30–40 min, during which the tube was inverted for a few seconds. Samples were centrifuged at 10,000× *g* for 10 min at 4 °C. Then, the supernatant was carefully transferred to a new 1.5 mL tube, 600 μL phenol/chloroform was added, and the sample was centrifuged at 10,000× *g* for 5 min at 4 °C. The supernatant was transferred to a new microcentrifuge tube and precipitated in 250 μL 8 M LiCl at −70 °C overnight. The tube was centrifuged at 10,000× *g* for 40 min at 4 °C. After centrifugation, the pellets were washed twice with 70% cold ethanol, dried, and dissolved in DEPC-treated distilled water.

### 4.5. cDNA Synthesis

Genomic DNA contamination was removed by treatment with DNase I (Takara Bio. Inc., Shiga, Japan) at 37 °C for 30 min. 500 ng RNA was used for cDNA synthesis, which was performed by reverse transcribing mRNA using the Clontech cDNA synthesis kit (Takara Clontech, CA, USA). cDNA synthesis was performed in the thermal cycler under the following conditions: 72 °C for 3 min followed by cooling the tube at 4 °C. The master mix was added to the pre-cooled tube, which was immediately incubated at 42 °C for 4 h. The reaction was terminated by heating at 70 °C for 10 min. Synthesized single-strand cDNA was stored at −20 °C until use.

### 4.6. Quantitative Real-Time-PCR (qRT-PCR) Analysis

Quantitative real-time PCR was performed using a CFX96 Real-Time PCR detection system (Bio-Rad Laboratories, Hercules, CA, USA) with the SYBR Premix (Toyobo, Osaka, Japan). The qRT-PCR mixture contained 5 nmol of each primer and the SYBR Premix. The reactions were run with the following cycle conditions: denaturation at 95 °C for 5 min, followed by 45 cycles of denaturation at 95 °C for 15 s and annealing at 60 °C for 30 s. Amplification of trehalase 1, GPI, and CS was performed with qRT-PCR primers. Primer pairs were designed using the PrimerQuest Tool (Integrated DNA Technologies, Coralville, IA, USA) and all primers used in the study are provided in Table 1. *M*. *incognita* β-actin (BE225475.1) primers were used as an internal control for normalization of gene expression. The 2^−ΔΔC^^t^ method of Livak and Schmittgen was used to quantify relative changes in gene expression levels [59]. Experiments were repeated three times with three independent biological samples. For mRNA expression analysis of RNAi transgenic lines, root gall samples were collected from infected lines, total RNA was isolated, cDNA was prepared, and real-time PCR analysis was carried out as described above. Primers used for qRT-PCR are provided in Table 4.

### 4.7. Plant Transformation and PCR Analysis

*Nicotiana benthamiana* was used to generate transgenic RNAi lines using the leaf disc method [60]. The generated CS-related cDNAs in the RNAi vector were transformed into the *Agrobacterium tumefaciens* strain EHA105. Regenerated plants were transplanted to rooting medium with kanamycin selection. Selective healthy transgenic lines were shifted to Soilrite pots for initial adaptation and further transferred to soil plants in a glasshouse. Putative transgenic lines were screened with BASTA selection as well PCR with gene-specific primers. Confirmed transgenic lines and control plants were grown in a glasshouse. Three consecutive generations of seeds were collected, and experiments were carried out with T3 transgenic lines. Experimental analysis of nematode hardening was carried out in a glasshouse.

### 4.8. RKN Inoculation and Suppression Analysis

The *M*. *incognita* resistance assay was carried out as previously described with few modifications [61]. To evaluate host-induced gene silencing of *M*. *incognita* chitin genes in tobacco, 2000 nematodes in stage J2 (suspended in liquid) were infiltrated into the soil surrounding the roots of each tobacco plant. Infected plants were grown in a greenhouse at 18–25 °C. At 45 days after infection, the roots separated from the plant and soil debris were removed by washing with tap water. Then, previously published methods [62] were used to count the numbers of eggs and J2, the number of roots with galls, and the number of galls, egg mass, eggs, and J2 number. RKN-infected roots were stained with erioglucine (100 mg/mL) for 15 min, and root knots and egg mass were counted. 

### 4.9. Statistical Analyses

All the experiments mentioned in this study had at least three independent biological and technical replicates. By default, the mean value of three replicates was considered for data analysis, and the standard deviation was presented as error bars in graphs drawn using SigmaPlot 12.5. The SigmaPlot 12.5 tool was used to perform *t*-tests to assess the significant variation that exists between control wild-type and RNAi transgenic lines. Statistics by *t*-test are shown, * *p* < 0.05, ** *p* < 0.001, and *** *p* < 0.0001.

## Figures and Tables

**Table 1:** List of primers used for cDNA cloning.

Name	Sequence (5′ to 3′)
**CS-F1-F**	ATGGTTAAAGGCCCCTCAACTG
**CS-F1-R**	TTATAAAAAAACCTGTGACCACC
**CS-F2-F**	ATGGTTAAAGGCCCCTCAACTG
**CS-F2-R**	CTCATAAAGTTCTTGAAAAAGACC
**CS-F3-F**	AAATATGGCATGAGAAAGCTCAATC
**CS-F3-R**	ATTCGAAGAGGGCTTTCCTCAG
**GPI-F1-F**	ATGACTTCAACAATTACTGGTCTA
**GPI-F1-R**	TCAATCCTTGTAATTTTTAATTAAATTA
**GPI-F2-F**	ATGCCTGATGTTAATGCTGTTC
**GPI-F2-R**	TCAATCTTTGTAATTTTTAATTAAATT
**GPI-F3-F**	ATGCCTGATGTTAATGCTGTTC
**GPI-F3-R**	CGTATGGTGAAGACC TCCAC
**TH1-F1-F**	ATGCTTTATTATGTTGTTTCTTTGC
**TH1-F1-R**	TTAAAATACATTATTTAAATAAATTCTTT
**TH1-F2-F**	ATGCTTTATTATGTTGTTTCATTGC
**TH1-F2-R**	TCAATTCATATGCACCATTGGAG
**TH1-F3-F**	TAATTGAAGGCTTCCGTACCAG
**TH1-F3-R**	TTAAAATACATTATTTAAATAAATTCTTT

**Table 2:** List of primers used for construction of the destination vector.

Name	Sequence (5′ to 3′)
Phos-gusf1	ATGTTACGTCCTGTAGAAACCCC
Phos-gusr1	TCATTGTTTGCCTCCCTGCTGC
RNAi UBQ1 F9	cggaattcAGGTGCCAAATCTTTGATTGGAGTTG
RNAi UBQ1 R12	aagcttCTTTTGTGTTTCGTCTTCTCTCACG
RNAi UBQ1 R13	ggtaccctcgagaagcttCTTTTGTGTTTCGTCTTCTC
RNAi UBQ1 R14	cggaattcggtaccctcgagaagcttCTTTTGTG
MCSF8	CCGAGCTCGCCCAAGCTTACGCGTGGATCCCTGCAG
MCSR8	CTGCAGGGATCCACGCGTAAGCTTGGGCGAGCTCGG

Restriction enzyme sites in primers are underlined and shown in lowercase letters.

**Table 3:** List of primers used for the destination vector.

Name	Sequence (5′ to 3′)
pK7-F	TTTGCGGACTCTAGCATGGCCGCG
Int-R1	CTTGAAAGTCAAATTGTCGAATTTG
Int-R2	GATCGGTGTGATACAAAACCTAATC
UBQ-F	CCATCTTAGACTTAGCTAAGTTT

**Table 4:** List of primers used for qPCR analysis.

Name	Sequence (5′ to 3′)
**Mi-actin F**	TTATTCTTTCACCGCAACCG
**Mi-actin R**	TTGACCGTCAGGCAATTCAT
**CS-F**	CACTTGTGCCTTTCACTGTTTC
**CS-R**	TGATGGTAGACTTGCGGTAATG
**GPI-F**	TGGCCAATGGACTGGTTATAC
**GPI-R**	TTGAGTGCTTCAGTGACCATTA
**TH1-F**	CAGAAGGGTAAAGGACGATGTT
**TH1-R**	AACGACCACCAGGAATGATAAA
**CS-RNAi-F**	CGTATTTGGAGACCAAGCAAAG
**CS-RNAi-R**	ACACTGGATGGATACACGTAAA
**GPI-RNAi-F**	TACTCCAAATACATTGGGCTCTT
**GPI-RNAi-R**	GCTAATTGTTTGCCTAATTCAACAC
**TH1-RNAi-F**	CCCTGGACATGAACTACAAGAA
**TH1-RNAi-R**	CCCAACGCCGAAGTTGATA

## Supplementary Materials

Supplementary Materials can be found at https://www.mdpi.com/1422-0067/21/18/6626/s1. Table S1. Category and name of chitin biosynthesis genes and proteins in Meloidogyne species. Table S2. Name and homology of chitin synthesis proteins putatively predicted from parasite nematodes. Table S3. Discrimination efficiency of transgenic plant using GUS staining. Figure S1. Predicted biosynthetic pathway from the M. incognita genome and number of genes involved in chitin biosynthesis of M. incognita. Figure S2. Confirmation of transgenic plants using GUS staining and PCR analysis.

## Abbreviations

RKN	Root knot nematode
J2	Juvenile 2
RNAi	RNA interference
qRT-PCR	Quantitative real-time polymerase chain reaction
UBQ	Ubiquitin
GPI	Glucose-6-phosphate isomerase
dsRNA	Double-stranded RNA
