# Supplementary material for: Chitin Biosynthesis Inhibition of Meloidogyne incognita by RNAi-Mediated Gene Silencing Increases Resistance to Transgenic Tobacco Plants
Source: Int J Mol Sci. 2020 Sep 10;21(18):6626. doi: 10.3390/ijms21186626 (PMC7555284; doi:10.3390/ijms21186626)
Supplement: Supplementary file 1 [file ijms-21-06626-s001.pdf]

International Journal of
Molecular Sciences

MDPI

Supplementary Data

Supplementary Table S1. Category and name of chitin biosynthesis genes and proteins in *Meloidogyne* species.

Enzyme	Number of genes (*C .elegans*)	Protein I.D. of *M. incognita*	Nematodes
Chitin synthase	T25g3.2<br>F48A11.10	Minc05474 (1)*<br>Minc05475 (1)<br>Minc05476 (2)	Mare1s00142g003945,<br>Mare1s00198g00230,<br>Mare1s04036g045576,<br>Minc3s00218g07846<br>Minc3s01800g26401<br>Minc3s0226g28968,<br>Mjav1s1194g063309
UDP-*N*-acetylglucosamine pyrophosphorylase	C36A4.4	Minc09215 (1)	Mare100298g007282,<br>Mare1s04620g049276,<br>Minc3s05172g37731,<br>Mjav1s05540g039915,
Phospho acetyl glucosamine mutase	F21D5.1.1	Minc10603 (1)	Mare1s01246g020988,<br>Mare1s139910g083231,<br>Minc3s00584g14658,<br>Minc3s06387g39757<br>Mjav1s01958g019618<br>Mjav1s04541g035120,
Glucosamine-6-phosphate *N*-acetyltransferase	T23G11.2<br>B0024.12	Minc12328 (1)	Mare1s100551g011625,<br>Minc3s00176g06824,<br>Minc3s02171g28677,<br>Majav1s02936g026178,<br>Majav1s05142g038039,<br>Majav1s09871g056965
Glutamine:fructose-6-phosphate aminotransferase	F07A11.2<br>F22B3.4	Minc11272 (1)<br>Minc07708a(2)	Mare1s08185g066263<br>Mare1s10030g072744,<br>Minc3s01527g24500<br>Minc3s03033g32504,<br>Mjav1s10530g059075,<br>Mjav1s20227g082278,
Glucose-6-phosphate isomerase	Y87G2A	Minc04129 (1)<br>Minc12596 (1)	Mare1s02668g035222,<br>Mare1s12192g078903<br>Mare1s15759g087264,<br>Minc3s00787g17359,<br>Minc3s07436g41129,<br>Mjav1s05090g037775,<br>Mjav1s11328g061484,<br>Mjav1s18665g0792
Hexokinase	F14B4-2<br>H25P06.1<br>Y77E11A.1	Minc08353 (1)<br>Minc17734 (1)<br>Minc09726 (2)<br>Minc19166 (2)	Mare1s03187g039510<br>Mare1s09608g071332<br>Mare1s01289g021462,<br>Minc3s00088g04155<br>Minc3s00699g16252<br>Minc3s01353g23041,<br>Mjav1s8007g050316,

			Mjav1s09188g054707,
Trehalase	F57B10<br>T05A12.5<br>W05E10.4<br>F15A2.2<br>C23H3.7	Minc05643 (1)<br>Minc05451 (1)<br>Minc12603 (1)<br>Minc12604 (1)<br>Minc05044 (2)<br>Minc10188 (3)	Mare1s01958g028694<br>Mare1s03269g040182,<br>Mare1s04702g049748,<br>Minc3s00333g10409,<br>Minc3s00692g16151<br>Minc3s02136g28499,<br>Mjav1s02658g024382,<br>Majv1s01624g017129

*Numbers in parentheses indicate proteins predicted the *M. incognita* genome.

Supplementary Table S2. Name and homology of chitin synthesis proteins putatively predicted from parasite nematodes.

Protein name	Protein ID	Size (amino acid)	Organism	E-value
Chitin synthase	Minc05474	556	*Meloidogyne incognita*	
	Minc05475	201	*Meloidogyne incognita*	1.37e^{-129}
	RCN35226.1	1268	*Ancylostoma caninum*	2e^{-103}
	AAG49219.1	1317	*Brugia malayi*	3e^{-102}
	NP_492113.2	1322	*Caenorhabditis elegans*	3e^{-101}
	KJH51876.1	2071	*Dictyocaulus viviparus*	8e^{-66}
	AAL92023.1	905	*Dirofilaria immitis*	1e^{-112}
	AAG39382	1356	*Dirofilaria immitis*	2e^{-98}
	XP_020305771	987	*Loa loa*	1e^{-68}
	PDM64072.1	1328	*Pristionchus pacificus*	2e^{-98}
	XP_024504714	1322	*Strongyloides ratti*	3e^{-74}
	EJW80710	829	*Wuchereria bancrofti*	1e^{-105}
Glucose- 6-phosphate isomerase	Minc04129	274	*Meloidogyne incognita*	
	Minc12596	206	*Meloidogyne incognita*	4.15 e^{-87}
	KJH49928.1	544	*Dictyocaulus viviparus*	2e^{-68}
	ADA56786.1	557	*Euphydryas aurinia*	2e^{-69}
	OTF83697.1	291	*Euroglyphus manynei*	4e^{-70}
	XP_003746506.1	551	*Galendromus occidentalis*	2e^{-68}
	XP_013777447.1	559	*Limulus polyphems*	6e^{-72}
	KHJ78041.1	196	*Oesophagostonum dentatum*	1e^{-69}
	XP_028165931.1	334	*Ostrinia furnacalis*	5e^{-72}
	XP_022818754.1	556	*Spodoptera litura*	2e^{-68}
	QQR67852.1	554	*Tropilaelaps mercedesae*	1e^{-68}
	XP_026483280.1	587	*Vanessa tameamea*	2e^{-68}
Trehalase 1	Minc05044	590	*Meloidogyne incognita*	
	Minc05643	460	*Meloidogyne incognita*	0
	AHM26075.1	571	*Anisakis simplex*	6e^{-29}
	XP_001896744	591	*Brugia malayi*	1e^{-36}
	EGT38784.1	567	*Caenorhabditis brenneri*	2e^{-24}
	NP_491890.2	567	*Caenorhabditis elegans*	2e^{-24}
	XP_003099055.1	568	*Caenorhabditis remanei*	6e^{-23}
	XP_003138812.1	567	*Loa loa*	8e^{-36}
	PDM77984.1	1238	*Pristionchus pacificus*	4e^{-23}
	XP_024505849	539	*Strongyloides ratti*	2e^{-23}
	KHN79069.1	1047	*Toxocara canis*	5e^{-28}
	KRZ05159.1	1966	*Trichinella zimbabwensis*	4e^{-20}

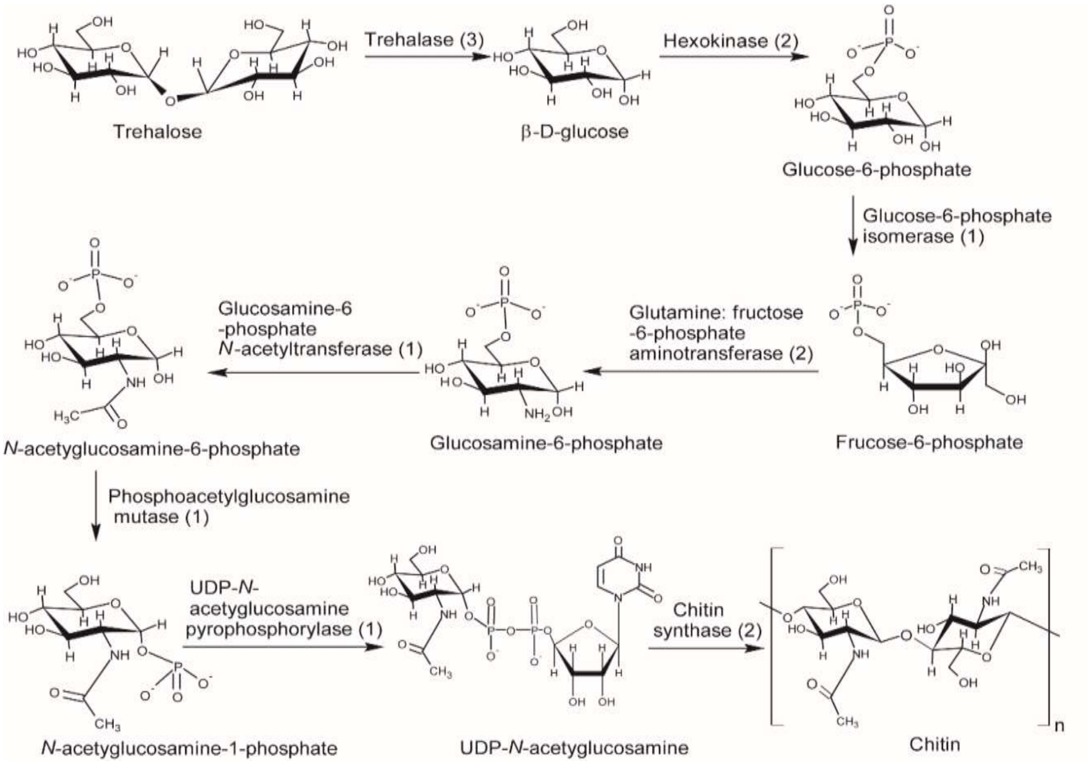

Supplementary Figure S1. Proposed biosynthetic pathway and number of genes involved in chitin biosynthesis of *M. incognita*. Trehalase-3 (Minc05643, Minc05451, Minc12603, Minc12604, Minc05044, and Minc10188); GPI (Minc04129 and Minc12596); and CS-2 (Minc05474, Minc05475, and Minc05476) are shown. The figure is modified from Hans Merzendorfer and Lars Zimoch [41]. The numbers in parentheses indicate proteins predicted from the *M. incognita* genome [42].

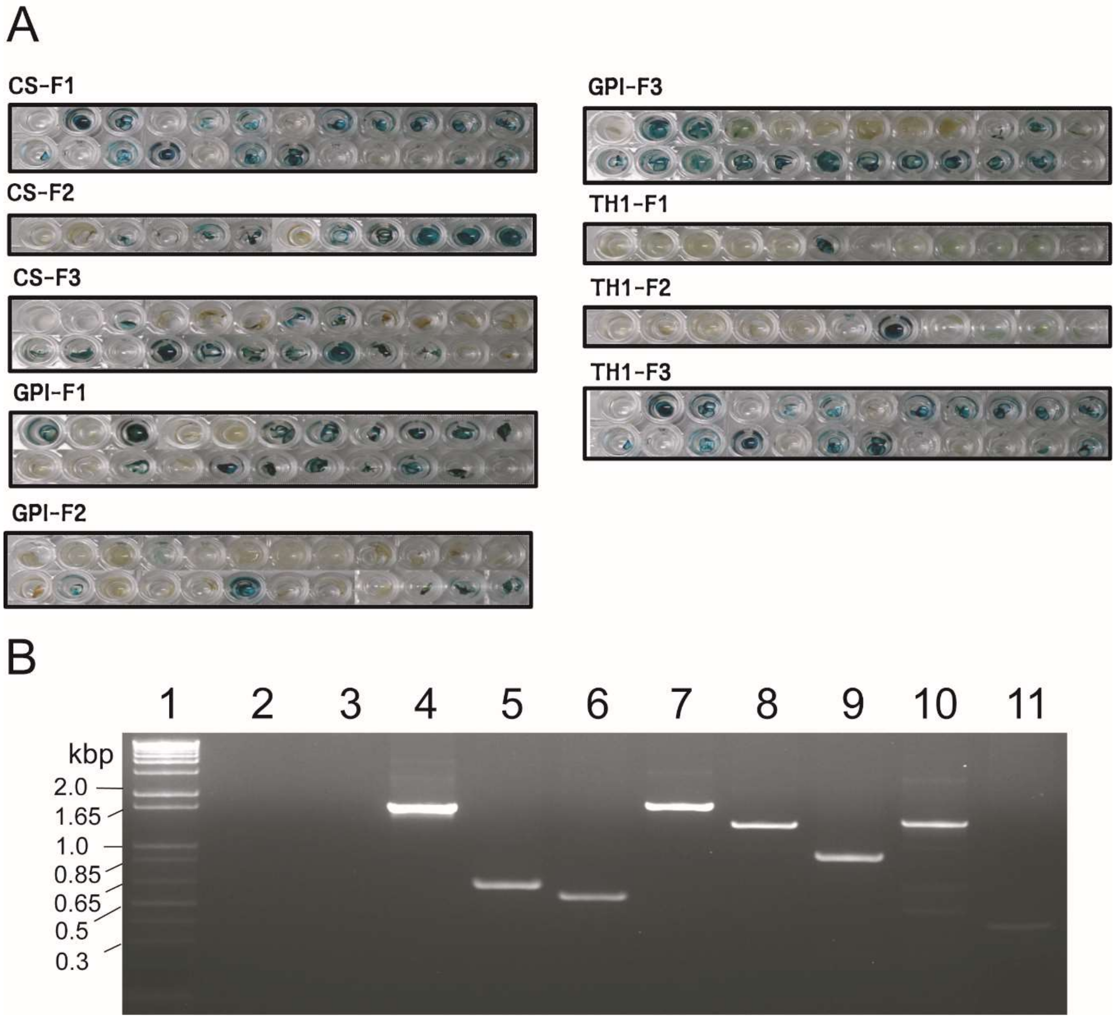

Supplementary Figure S2. Confirmation of transgenic plants using GUS staining and PCR analysis. (A) GUS staining of selected transgenic plants. (B) PCR analysis of selected transgenic plants. Genes are chitin synthase (CS), glucose-6-phosphate isomerase (GPI), and trehalase (TH). Lane 1, molecular standard markers; lane 2, wild type; lane 3, pBSGW; lane 4, CS-F1; lane 5, CS-F2; lane 6, CS-F3; lane 7, GPI-F1; lane 8, GPI-F2; lane 9, GPI-F3; lane 10, TH1-F2; and lane 11, TH1-F3.

Supplementary Table S3. Discrimination efficiency of transgenic plant using GUS staining.

Gene	No. of regenerated plant	No. of gus stained plant	Ratio (%)
CS-F1	68	22	32.4
CS-F2	57	25	43.9
CS-F3	77	33	42.9
GPI-F1	50	16	32.0
GPI-F2	88	17	19.3
GPI-F3	43	26	60.5
TH1-F1	15	1	6.7
TH1-F2	39	17	43.6
TH1-F3	58	36	62.1

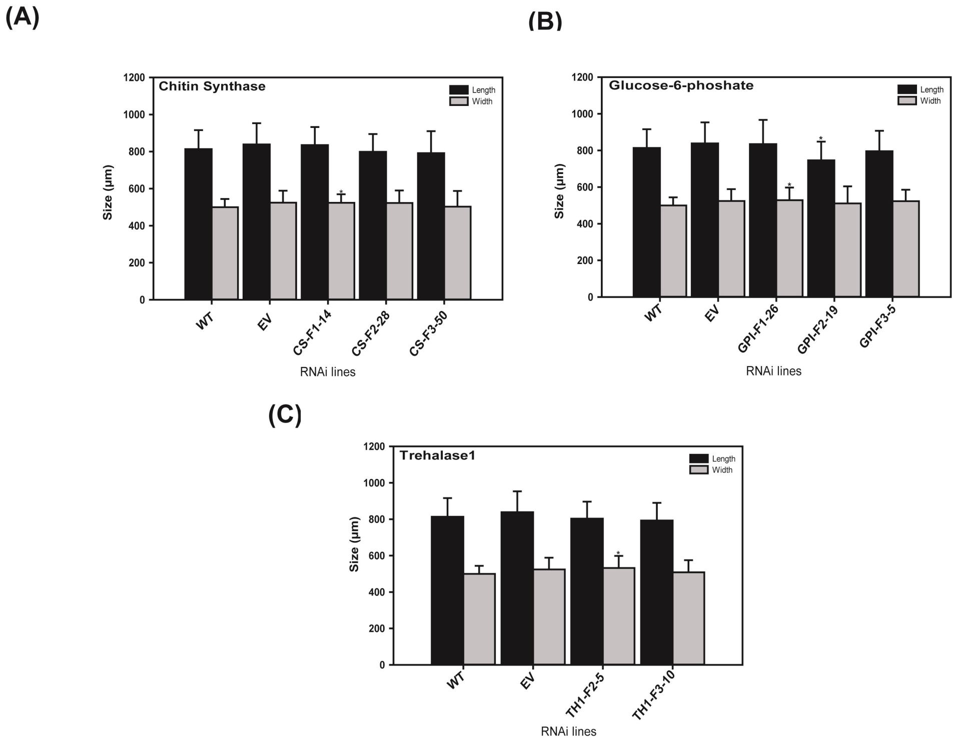

Supplementary Figure S3. Silencing of chitin biosynthesis genes affected width and length of female *Meloidogyne incognita* feeding on transgenic *Nicotiana benthamiana* plants. (**A**) CS RNAi lines. (**B**) GPI RNAi lines. (**C**) Trehalase RNAi lines are shown. Results are presented as mean ± standard deviation (SD) of three independent experiments with three technical replicates. Statistical analyses (t-test) were conducted in (Sigmaplot 12.5). For t-test analysis, WT data were used to compared with each RNAi lines. Asterisk indicates significant differences compared with wild type (WT).
